# Dip-Coating Fabrication of All-Polymer Multilayer Photonic Crystals through 3D Printer Conversion

**DOI:** 10.1021/acsapm.4c04077

**Published:** 2025-04-09

**Authors:** Martina Martusciello, Coralie Hervieu, Daniela Di Fonzo, Andrea Lanfranchi, Paola Lova, Davide Comoretto

**Affiliations:** † Dipartimento di Chimica e Chimica Industriale, Università di Genova, via Dodecaneso 31, 16146 Genova, Italy; ‡ École d’Ingénieurs Publique du MESRI, Sigma Clermont, 20 Avenue Blaise Pascal, TSA 62006, 63178 Aubiere Cedex, France

**Keywords:** dip-coating, polymer thin films, photonic crystals, 3D printer conversion, multilayer coatings, distributed Bragg reflectors, automated fabrication, scalable photonics

## Abstract

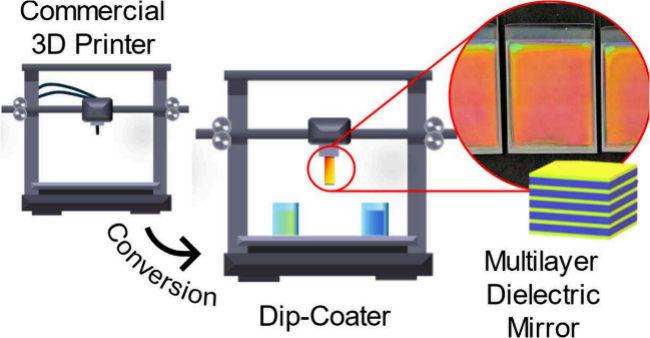

Dip-coating plays a crucial role in the production of polymer thin films and coatings on both laboratory and industrial scales. The simplicity of the process, combined with its adaptability and precision, makes it an invaluable technique for achieving consistent and reproducible coatings, which can also be suitable for photonics applications. As a cheaper, more flexible alternative to commercial dip-coaters, we report on the conversion of a commercial 3D printer designed for fused deposition modeling into a dip-coating system for fabricating multilayered photonic crystals. The feasibility of this approach is demonstrated by fabricating both distributed Bragg reflectors and a fluorescence planar microcavity. We used a perfluorinated polymer formulation and poly(*N*-vinylcarbazole) as structural dielectric media, alongside a recycled blend of fluorescent polystyrene as a light emitter for the microcavity. In both cases, precise control of the deposition parameters enables the formation of uniform photonic nanostructures, leading to a spectral redistribution of fluorescence comparable to that achieved by standard spin-coated photonic crystals. This approach paves the way toward automating the fabrication of planar photonic structures on a laboratory scale, with the potential to scale up to larger surface areas compared to those obtained by standard methods.

## Introduction

**Figure 1:**
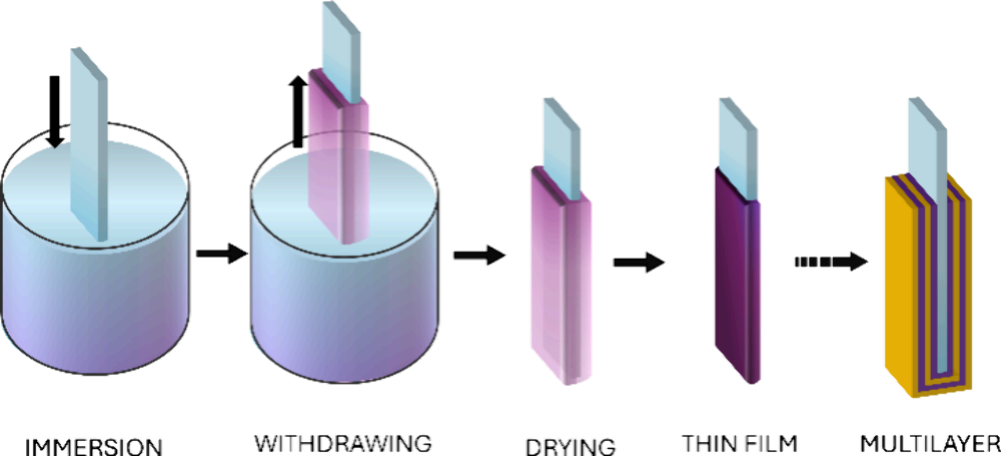
Schematic of the dip-coating process.

Dip-coating is a widely used and versatile manufacturing process for the production of thin films and coatings.^1^ In this process, an object or a substrate is immersed in a liquid solution, often referred to as a coating material or dip solution, and then removed in a controlled manner (Figure 1).^2^ The evaporation of solvent from the liquid’s meniscus results in a uniform layer of material that adheres to the surface of the substrate.^3,4^ This method is suitable for casting films made of various materials including polymers,^5,6^ ceramics,^7^ and composites,^8^ enabling the production of coatings with customized properties such as optical transparency, electrical conductivity, corrosion resistance, and more.^9^ In the case of single-layer deposition, dip-coating represents a valuable technique for achieving reproducible coatings.^10^ However, when multilayer structures are involved, control over film thickness, roughness, and uniformity becomes stricter. This is the case with photonic crystals, including distributed Bragg reflectors (DBRs) and planar microcavities, which are of paramount technological importance in photonics to control light propagation, emission, and light–matter interaction.^11−15^ In this regard, there are few reports on the fabrication of inorganic and polymer-inorganic hybrid systems by dip-coating in the literature, whereas limited emphasis is paid to polymers.^16−19^

DBRs’ manufacturing is based on the alternate deposition of thin films^20,21^ to obtain a periodical modulation of the refractive index with a pitch on the order of the optical wavelength.^22^ Such modulation causes coherent diffraction phenomena, allowing for the formation of photonic band gaps (photon bandgaps). These are energy regions where light propagation in the structure is forbidden and impinging light is reflected, making the structure behave as a dielectric, lossless mirror. Typical examples of photonic structures observed in nature are chameleons, peacocks, or morpho butterflies, characterized by vivid, iridescent colors that are due to the presence of photon bandgaps in the visible range.^22,23^ Indeed, each photon bandgap can be detected in the reflectance spectra of DBRs and microcavity as a peak whose intensity depends on the refractive index difference between their components and on the number of layers composing the structure. For these reasons, DBRs have applications in various research and technology areas such as sensing,^24−27^ laser,^12,13^ thermal shielding,^28^ and smart packaging.^24,29^ Microcavities, on the other hand, represent a modification of the DBR architecture in which the periodicity is broken by a “defect” layer, enabling the propagation of some wavelengths within the photon bandgap spectral region.^11,30^ This effectively means having the defect layer sandwiched between two lossless mirrors, preferable to metallic ones for photonic applications.^31^ Typically, a fluorophore is embedded in the defect layer to modify the spontaneous emission. Indeed, when the structure is properly engineered, spectral fluorescence redistribution and changes in its radiative rate can be observed.^14,30,32,33^

Usually, spin-coating is the technique of choice for the fabrication of polymer DBRs and microcavities at the laboratory scale.^15^ In this technique, a solution is cast onto a rotating substrate.^34^ Centrifugal forces spread the solution evenly while the solvent evaporates, resulting in homogeneous films of controlled thickness. However, spin-coating is usually manual, which makes it highly time-consuming and very sensitive to different operators. It is therefore worth considering dip-coating as a promising alternative to improve automation and repeatability.^15^ The dip-coating process is governed by a delicate balance of factors, including the viscosity of the solution, the speed of substrate dipping, drying conditions, and substrate properties, which are particularly severe when strict thickness uniformity and control are required.^3^ As the substrate is drawn out of the solution, a combination of capillary forces, gravity, and fluid dynamics influences dip-coating, resulting in a complex deposition process.^3^ As a consequence, several chemical and physical parameters must be considered: concentrations of the solutions, orthogonality of the solvents used to cast the subsequent layers, withdrawal velocities (*v*), and drying time (*t*). For this reason, dip-coaters are often rather expensive machines coming with very limited degrees of freedom.^35,36^

Rauh et al.^37^ provided a quite thorough work on the conversion of an FDM 3D printer into a dip-coater. They optimized it to attain a low-cost alternative for the deposition of single poly(methyl methacrylate) films. Their findings showed that 3D printers can be efficiently converted into coating systems, at least for single films. Henceforth, we made progress by enabling the deposition of different materials on the same substrate, resulting in high-quality multilayered structures with subnanometric surface roughness from technical polymers, providing a fully automated alternative to spin-coating. At the hardware level, the conversion is performed through the simple replacement of original components with 3D-printed plastic parts; at the software level, a MatLab application is developed to write easy dip-coating routines for the printer to follow.^38^ The 3D printer’s extruder head is replaced with an opportunely designed clip-like holder for the substrate, allowing precise three-axis movement. The conversion allows one to dip the substrate in two or more solutions in a controlled manner, with a reduced cost compared to commercial dip-coaters with a similar multilayer functionality. In principle, the 3D printer even provides the possibility to heat the solutions to a controlled temperature by heating the building plate. To demonstrate the feasibility and success of the approach, we show the fabrication of different polymer multilayers made of poly(*N*-vinylcarbazole) (PVK, high refractive index; *n* = 1.68)^28^ and Aquivion (AQ, low refractive index; *n* = 1.34),^28^ a perfluorinated ionomer material. The optical properties of the dip-coated samples are comparable to state-of-the-art spun-cast samples.^29^ For the microcavity defect layer, a polystyrene derivative obtained from a recycled ice cream spoon was used as the fluorescent material. Microcavities require controlling the thickness of the layers to tune the position of the photon bandgap. Therefore, the fabrication of a microcavity bearing adequate optical response is notoriously more difficult than a simple DBR and a significant stress test for the process, demonstrating its feasibility.

## Experimental Methodology

### Dip-Coating Apparatus

**Figure 2:**
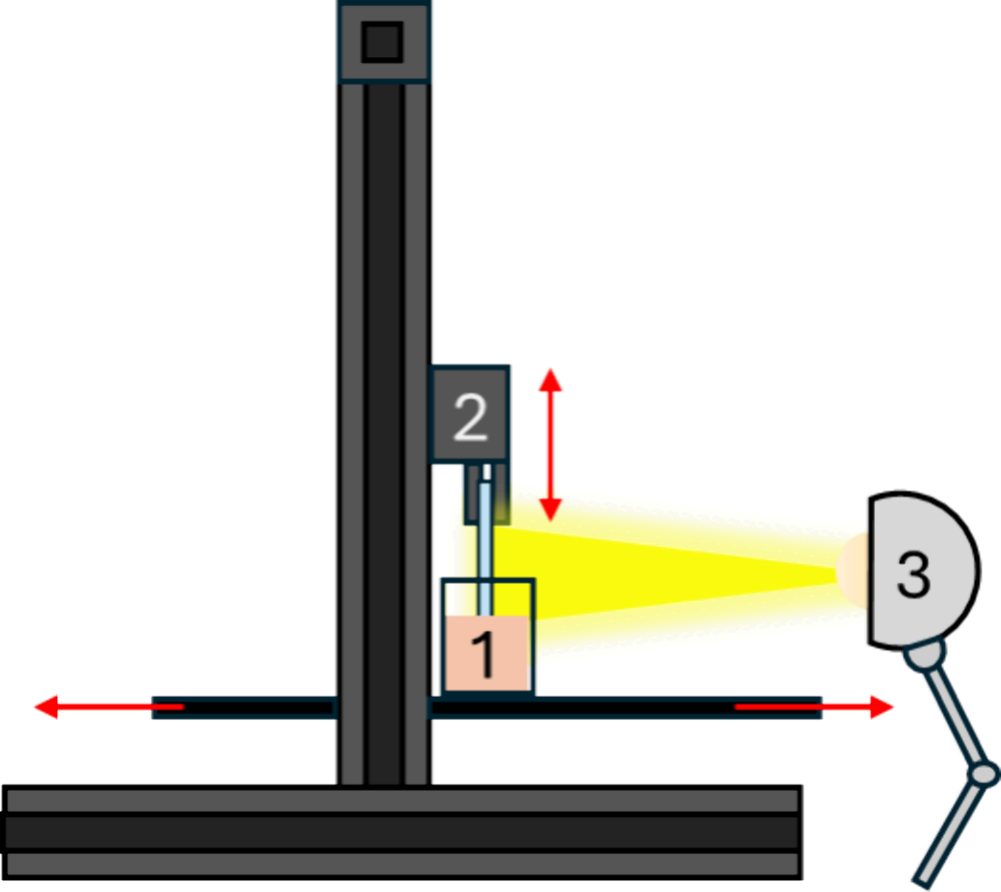
Schematic of the dip-coating apparatus consisting of an FDM 3D printer (Creality Ender 3-V2) optimized with a 3D-printed clip (2), beaker containing the polymer solution (1), and an IR lamp, placed 20 cm away from the sample (3).

The apparatus is schematically represented in Figure 2, whereas a digital photograph of it is shown in the Supporting Information Figure S1. It consists of an FDM 3D printer (Creality Ender 3-V2) optimized with a 3D-printed clip replacing the extruder to firmly hold the 25 mm × 75 mm × 1 mm glass substrate (Figure 2, inset 2). The containers for the dipping solutions consisted of two polypropylene beakers with 3D-printed nylon caps with rectangular open slits to allow dipping of the substrate into the container (element 1 in Figure 1). The caps minimize the evaporation of the solvents during the process, thus reducing the variation of polymer concentration in the casting solutions. During the coating, the containers were placed in marked positions on the printer’s building plate and filled with adequate polymer solutions/dispersions. The machine alternatively dipped the substrate in each solution, withdrawing it at a constant preset speed, with a preset drying time before switching solutions. IR lamps with different powers were used during the film drying process (element 3 in Figure 1), placed around 15 cm from the sample. To allow the 3D printer to move the substrate according to the dip-coating needs, a custom MATLAB application was developed. The application converts the user inputs (withdrawal speeds of the substrate from each of the solutions and waiting time between layers for drying) into a proper G-code file containing the instruction for the 3D printer. Afterward, the G-code is loaded onto an SD card and read by the printer as a standard printing file to be executed.

### Sample Fabrication

The setup was used to fabricate various DBRs and microcavities at room temperature. All samples were prepared on glass substrates with a thickness of 1 mm and a width of 25 mm. The length of the sample ranged from 15 mm to 30 mm depending on the dipping depth. DBRs were fabricated via dip-coating of an aqueous dispersion of AQ (DB79, Solvay Specialty Polymers, *n* = 1.34)^28^ diluted 1:10 in EtOH alternated with a solution of PVK (AcrosOrganics, *M*_w_ = 156,000, *n* = 1.68)^28^ in toluene (Sigma-Aldrich, ACS ≥ 99.5%) with concentration 10 mg mL^–1^. Different withdrawal speeds were tested (1, 2, 4 mm s^–1^) to fabricate DBRs made of 5.5 layers (11 layers; schematically 5 PVK-AQ layer pairs plus a capping PVK one on top, hereafter indicated as ((PVK-AQ)^5^PVK). Commercial infrared lamps of different powers (50, 100, and 250 W; Trixie Reptiland 50 and 100 W and Philips Incandescent BR125 250 W) were tested for the film drying process during the fabrication of DBRs of 3.5 bilayers each. Each layer was dried and irradiated for 3 min. Repeatability experiments were performed by fabricating DBRs with 4.5 BL each. The AQ dilution in ethanol, the PVK concentration, and the withdrawal speed for each sample were, respectively, 1:20, 10 mg mL^–1^, and 3 mm s^–1^ for S1; 1:20, 10 mg mL^–1^, and 4 mm s^–1^ for S2; and 1:10, 11 mg mL^–1^, and 6 mm s^–1^ for S3. Note that the AQ batch used for the repeatability experiment is different from the first one; hence, the different operating conditions attain similar results.

The microcavity consisted of a (PVK-AQ)^5^ DBR, a single defect layer, and an (AQ-PVK)^5^ DBR on top, deposited sequentially. DBRs were fabricated with the aforementioned method, whereas the defect layer was deposited via dip-coating the (PVK-AQ)^5^ DBR in a 1 mg mL^–1^ solution of a styrene-based xanthenic dye obtained by directly dissolving a piece of an ice cream spoon in toluene (represented in Supporting Figure S2).^39−41^ Since dip-coating deposits symmetrically on both sides of the glass substrates, DBRs are effectively doubled; to avoid complex light confinement effects, the microcavity is instead removed from one glass side.

### Thermal Analysis

The thermal video was shot at 7.5 Hz framerate with an FLIR T560 thermal camera working with a germanium lens and a 24° viewing angle (resolution 640 × 580 pixel, accuracy ± 2 K, sensitivity <40 mK @ 30 °C). The video was then analyzed with the FLIR Research Studio software.

### Atomic Force Microscopy

Atomic force microscopy was performed on a dip-coated sample (Supporting Information S2a) as well as on a spin-coated sample with the same number of layers and a similar spectrum, fabricated from a 30 mg mL^–1^ PVK solution and 1:10 AQ solutions, cast at 100 rotations per second. The micrographs were taken in the dynamic force mode in air, using a Nanosurf FlexAFM microscope on an area of 10 μm × 10 μm. Analyses of the scans were performed using the open-source software Gwyddion.

### Optical Characterization

Normal incidence reflectance measurements were recorded with a custom fiber optic setup consisting of deuterium and tungsten halogen sources (spectral range of 230–2500 nm) using the reflectance of a UV-enhanced aluminum mirror or a protected silver mirror as a reference (Thorlabs). The reflected signal was recorded with an AvaSpecULS4096CL-EVO spectrometer (spectral range 250–1100 nm, resolution 1.4 nm) for the visible range and an AvaSpec-NIR (spectral range 910–1700 nm, slit size 50 μm) for the near-infrared range. Angle-resolved transmission and photoluminescence spectra were recorded using a home-built setup with an angular resolution of 2° using the same light source and spectrometer. The steady-state PL measurements were performed by exciting the samples with a 405 nm CW laser MatchBox series focused on a 1 mm spot. The signal was collected with a parabolic mirror and then detected with the spectrometer. The collection setup allowed for the measurement of transmission and photoluminescence at the same spot. Photoluminescence decay was recorded using a PicoQuant time-correlated single-photon counting system (Time Harp 260 PICO board with a temporal resolution of 150 ps, a PMA Hybrid 40 detector, and a 405 nm optical fiber-coupled LDH-P-C-405 laser (PicoQuant, Berlin, Germany) with a PDL 800B driver with a 5–80 MHz repetition rate as the excitation source).

Fourier transform infrared spectroscopy was performed on a thick film of the same material used as the defect layer in the microcavity using a Bruker (Vertex 70) operating in the ATR mode (diamond prism) in the range of 400–4000 cm^–1^. All of the measurements were carried out at room temperature. The films were made by drop-casting 0.5 mL of the same solutions used to prepare the microcavity defect layer on a glass substrate.

## Results and Discussion

To optimize the custom deposition setup, we focused on substrate withdrawal speed and the drying condition using infrared lamps with different irradiation powers as the heating sources. Figure 2 shows a schematic of the complete apparatus.

### DBR Fabrication

**Figure 3:**
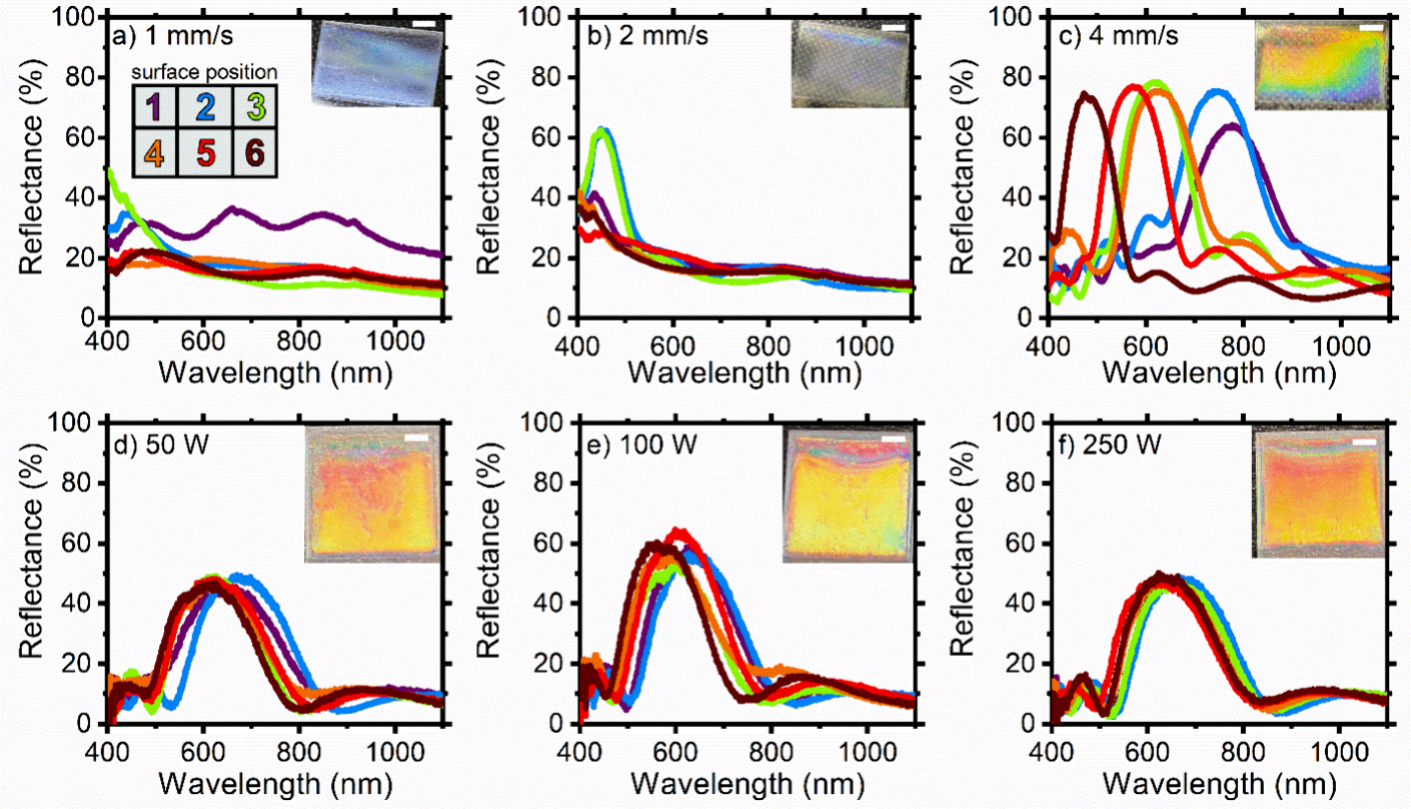
Reflectance spectra of the multilayers fabricated at withdrawal speeds of (a) 1, (b) 2, and (c) 4 mm s^–1^ and of samples fabricated at a withdrawal speed of 4 mm s^–1^ under infrared irradiation of (d) 50, (e) 100, and (f) 250 W. The white line is 5 mm.

We initially cast three samples made of 5.5 bilayers using different withdrawal rates (1, 2, and 4 mm s^–1^) without any heating source. Figure 3a shows the reflectance spectra collected in six different points on the surface for the multilayer obtained at a withdrawal speed of 1 mm s^–1^. In this sample, it is impossible to discern features that can be assigned to the presence of a photonic structure. However, all of the spectra show the presence of an interference pattern visible as an oscillation of the reflectance spectrum background, suggesting a discrete optical quality of the overall film obtained. The slight increase of the background value moving toward the short wavelength region is due to the increase in the refractive index of the PVK.^28^ The lack of characteristic peaks in the reflectance spectrum is also testified to by the digital image of the sample (inset) showing an opaque bluish color. On increasing the withdrawal speed to 2 mm s^–1^ (Figure 3b), the optical quality of the sample improves, especially in the upper part of the sample. Indeed, the spectra recorded in positions 2 and 3 show the presence of a photon bandgap centered at 448 nm with the reflectance larger than 60%. However, the sample was largely inhomogeneous, with the remaining part of the surface also easily recognizable in the digital image of the sample. Finally, in the sample cast with a speed of 4 mm s^–1^ (Figure 3c), all of the spectra show well-defined high reflectance peaks assigned to the photon bandgap. This result confirms the formation of a dielectric lattice. However, these peaks are positioned in a rather wide spectral interval (from 470 to 800 nm), demonstrating the impossibility of casting films with homogeneous thickness under these conditions. Indeed, the wavelength of the photon bandgap is directly proportional to the layer thickness, suggesting that it varies over the surface of the sample.^15^ Such inhomogeneity is also clearly detected in the digital photograph of the sample, which shows vivid and brilliant color shifting from blue to red on moving from position 6 to position 1.

Data discussed thus far also suggest that film thickness increases with the withdrawal speed. Indeed, for larger speeds, the photon bandgap is detected in the visible and near-infrared spectral ranges, while it moves toward the blue spectral region at lower speeds, and it cannot be detected anymore for the slowest withdrawal process. Referring to the dip-coating theory,^3,10^ during the withdrawal phase, various forces interact. These forces fall into two categories: draining forces and entraining forces. Draining forces pull the liquid away from the substrate and back into the bath, thus opposing the film deposition, while entraining forces keep the fluid on the substrate, favoring film formation. The equilibrium between these forces dictates the existence of two different regimes, where thickness decreases upon the increase of withdrawal speed when capillary entraining forces dominate or increases when draining forces dominate.^42^ The theory therefore suggests the setup is working in the draining regime, which seems confirmed by the results reported by Rauh et al. for comparable withdrawal speeds.^37^ Inhomogeneities due to the different layer thicknesses can be instead linked to the accumulation of solutions along the slow drying process.

Therefore, for speed drying, an infrared light source was used to heat up the sample during the withdrawal process. In this case, we tested three infrared lamp powers (50, 100, and 250 W), maintaining a constant withdrawal speed of 4 mm s^–1^ and 3.5 bilayers. Starting from the lower value of the power (50 W) (Figure 3d), it is possible to recognize the presence of a rather intense photonic band gap between 605 and 675 nm. There is also a good overlap of the spectra collected at the different points of the sample, despite some inhomogeneities. These characteristics are also confirmed by the digital image in the inset. The sample has two main color regions, indicating that, although homogeneity improved, it is still not satisfactory. Therefore, the power was increased to 100 W (Figure 3e). The optical response of the new sample shows a photon bandgap in the range between 564 and 633 nm. While homogeneity seems not to be affected by the larger lamp power, the intensity of the photon bandgap reflectance peak increased with respect to the previous case. This phenomenon prompted us to further increase the power of the lamp to 250 W (Figure 3f). The spectra collected at six points of the samples show a photonic band gap in the range between 628 and 662 nm, with a spectral shift of ≈30 nm. This is the most homogeneous sample, suggesting that a higher power of the lamp optimizes the drying conditions. The digital image shows a sample with a reddish uniform color interrupted by a yellowish shade at the bottom, only. The reproducibility of the process was tested by fabricating three different samples, in three copies each, as reported in the Supporting Information Figure S3a–c. The samples show an extremely similar appearance and spectrum; by changing the concentration of AQ and PVK and slightly changing the withdrawal speed, we were able to obtain reproducible samples showing the main photon bandgap from 620 (Figure S3a) to 1250 nm (Figure S3b) and to 1600 nm (Figure S3c). Note that the discontinuity between spectra at 900 nm observed in the plots is due to the use of different spectrometers for the visible and near-infrared ranges, as discussed in the Experimental Section. The samples with bandgap up to 1250 nm are quite uniform in appearance and spectrum over the whole area, whereas the one more toward the high-end of the near-infrared region is less homogeneous. This is unsurprising and often observed with large-wavelength bandgap AQ-PVK structures even in spin-coating.^28^ In addition to demonstrating the good reproducibility of samples produced via the method presented here, these results show that the method can produce a broad gamma of photonic structures, from the visible to the telecommunication near-infrared range.

### Surface Roughness Measurements

Surface roughness of a DBR produced via dip-coating was compared to that of the one obtained through spin-coating via atomic force microscopy, as per the experimental section. The 3D surface profiles are reported in the Supporting Information Figure S4a (for the dip-coated sample) and in Figure S4b (for the spin-coated sample). The root-mean-square roughness was around 0.8 nm for the dip-coated sample against 0.3 nm for the spin-coated one. The order of magnitude of roughness of the two samples is the same (and extremely low), and as such, it carries no observable negative effect on the spectra resulting from scattering. Indeed, this roughness is compatible with the fabrication of polymer DBRs as reported in the literature for spin-coated samples.^43^ Note that the spectrum and picture of the spin-coated sample are reported in Figure S4c.

### Thermal Analysis

**Figure 4:**
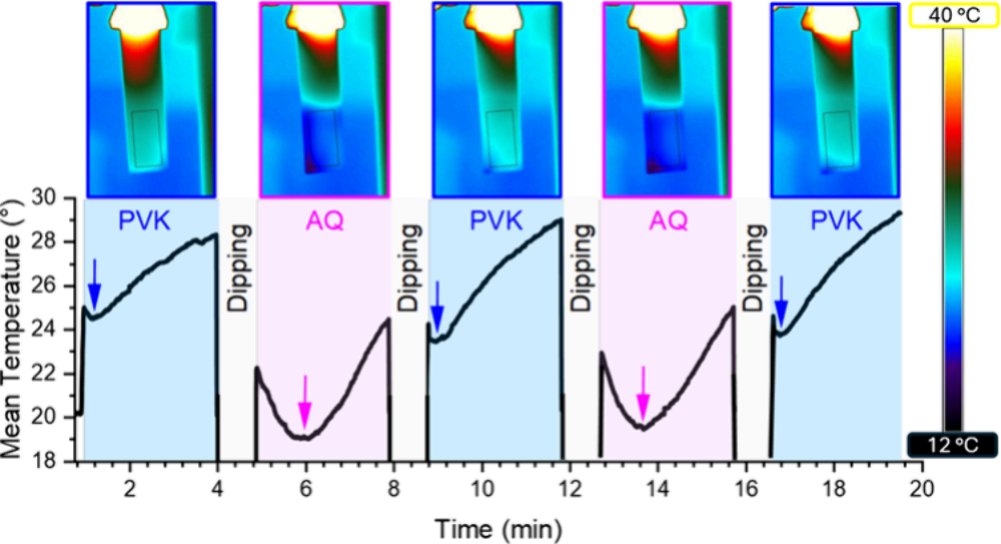
Temperature of the sample during the dip-coating of 2.5 BLs: frames of the thermal video (top images) and average sample temperatures (bottom graphs). Blue shades highlight the drying process for the PVK layers, and pink shades are for the AQ ones. The frame of the thermal video corresponds to the minimum temperature value. The black rectangle highlights the region of interest of the sample, where the average temperature is calculated. White gaps represent the dipping motion when the sample cannot be observed by a thermal camera.

To investigate the temperature reached by the samples during the drying process, we measured it via thermal imaging as discussed in the Experimental Section. We filmed the fabrication process of the first 2.5 BLs of a sample. We report the full video in the Supporting Information and the selected frames in Figure 4. The temperature is averaged over the black rectangle reported in the thermal frame, and its trend over time is reported in the plot. In the latter, the blue shades highlight the temperature profile during the drying process of PVK films, whereas the pink shades represent the drying process of AQ. Between them, the samples are dipped in AQ or PVK, respectively, and the movement of the gantry impedes the measurement. The overall behavior is first a decrease in the temperature up to a minimum, followed by a steady increase. The thermal frames reported correspond to the respective minima. There is a striking difference in the minimum temperature observed in the two cases, as immediately seen by the thermal images. Indeed, this value is around 24.5 °C for PVK and 19 °C for AQ. Moreover, from the curve, the duration of the temperature decrease is more long-lasting and pronounced for the AQ layers. The explanation for the difference presumably lies in the different latent heats of evaporation of the solvents making up the two polymer solutions. Indeed, toluene possesses a much lower enthalpy of evaporation (∼400 J g^–1^)^44^ with respect to ethanol (∼900 J g^–1^).^45^ This makes the evaporation of toluene so fast that the drop in temperature of the sample happens mostly before the sample is in the region of interest of the thermal camera and, hence, the short dip in temperature. On the other hand, the evaporation of ethanol is slower and requires more energy and time, lowering the temperature more and making the phenomenon more observable. A slight asymmetry in the evaporation process can be deduced from the thermal images, with the right-hand side of the sample being slightly hotter. This is probably due to the asymmetrical emission of the lamp. The information reported here is quite valuable, as when the temperature starts rising after the drop, it is presumably “safe” to dip the substrate in the other layer, as all of the solvent should be evaporated. This allowed us to fine-tune the drying times of the two layers.

### Microcavity Fabrication

As a proof of concept for more severe tests on the quality of the deposition systems, beyond the simple color uniformity, we fabricated and characterized a microcavity structure whose properties critically depend on the quality and reproducibility of the composing films (layer thickness homogeneity, small interface roughness, etc.).^30^ To demonstrate the efficacy of the system even when less-than-ideal materials are used, the fluorescent layer of the microcavity was made out of a recycled ice cream spoon, represented in the picture in Figure S2. The spoon looks green under standard illumination and fluoresces to a bright cyan color when excited with violet light. The spoon’s composition was verified via infrared spectroscopy; its spectrum is reported in the Supporting Information Figure S5 alongside the spectrum of polystyrene. The sample exhibits aliphatic and aromatic C–H stretching peaks at 2924 and 2851 cm^–1^ and at 3026 and 3082 cm^–1^, respectively. Around 1605 and 1585 cm^–1^, we observe aromatic C=C stretching vibrations. In the 1490–1450 cm^–1^ region, two sharp peaks are related to the bending vibrations of the aromatic C–H bonds. These correspond to the distinctive signals of polystyrene. We do not precisely know the composition of the xanthenic dye and whether it is dispersed or bonded to the matrix; we assume it is fluorescein or a related compound due to its color, low price, and food compatibility. However, the exact verifications would require a deep investigation quite beyond the scope of the paper.

**Figure 5:**
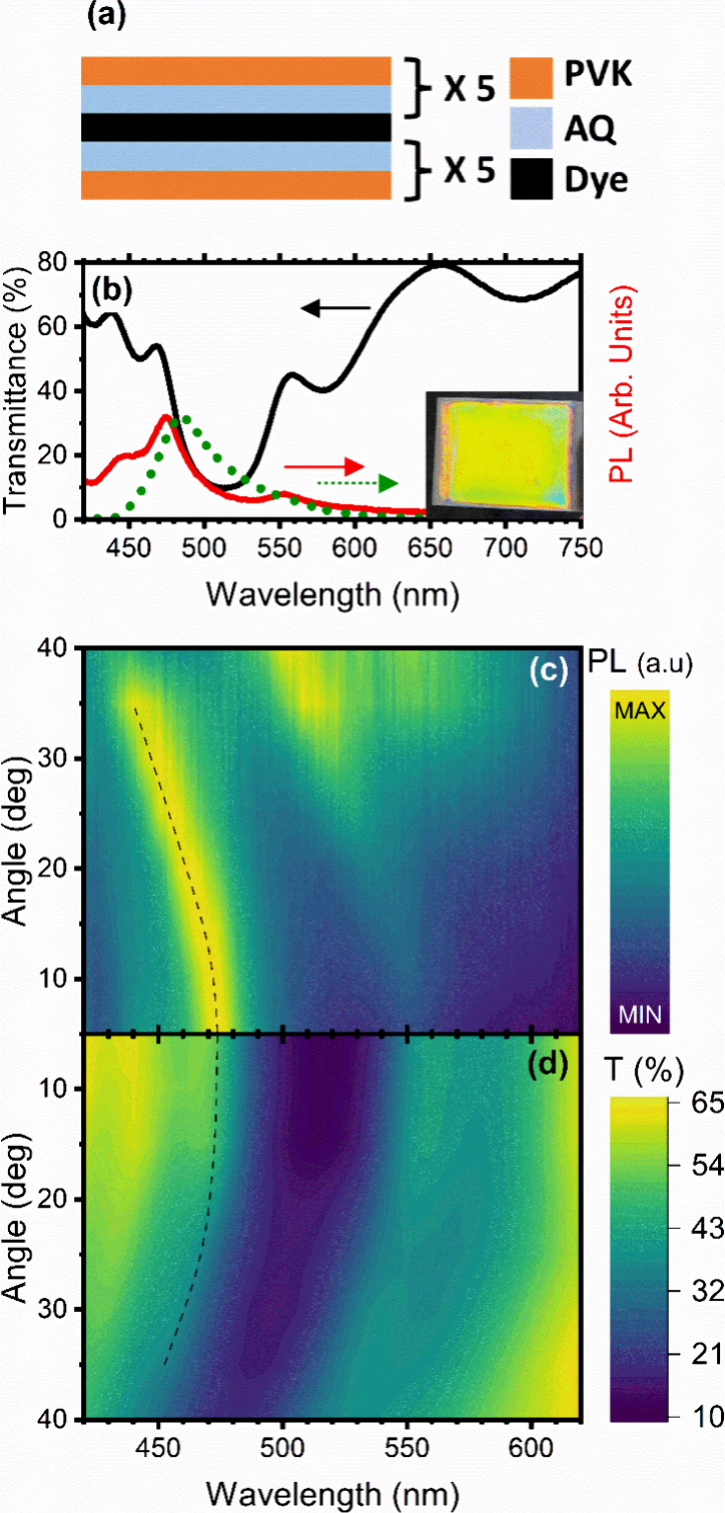
(a) Schematic of the microcavity. (b) Transmittance (black line) and PL (red line) spectra of the microcavity compared with the PL spectrum of a dye thin film (dotted green line). The inset shows a digital photograph of the sample. (c and d) Angle-resolved PL (c) and transmittance spectra (d) of the microcavity sample. The dotted line follows the cavity mode as a guide for the eye.

The microcavity was formed by two DBRs (PVK-AQ)^5^ as schematized in Figure 5a, sandwiching the PS-dye defect layer in the middle. The reflectance spectra of the first DBR as well as its uniformity are proven by the spectra reported in Supporting Figure S6, clearly showing the photon bandgap at 603 nm. The microcavity shows a brilliant green color visible in its digital image (inset of Figure 5b), with minor inhomogeneities on the edges where the border of the glass substrate affects the film deposition. Referring to Figure 5b, which shows the transmittance of the microcavity as a black line, the photon bandgap is detected as a broad low-transmittance region between 400 and 600 nm (the complementary reflectance, where the photon bandgap is detected as a high-reflectance region, is shown as a black line in Supporting Figure S7). Within the photon bandgap, we observe two relative transmittance maxima at 470 and 550 nm, most likely due to the presence of two cavity modes.^30^ The microcavity structure deeply modifies the density of local photonic states of the system and then the emission of the layer. This, in turn, has a strong effect on the fluorescence spectrum of the dye.^14^ In order to demonstrate this effect, in Figure 5b, we report as a dotted green line the fluorescence spectrum of a thin film of the PS-dye blend cast by using the same condition employed for the cavity. The spectrum shows an asymmetric, intense, and broad spectrum peaked at 480 nm. On the other hand, the microcavity, in agreement with theory,^11^ shows a severe spectral redistribution of its fluorescence (red line in Figure 5b). Indeed, in correspondence to the photon bandgap, the signal is almost completely suppressed, while in correspondence to the cavity modes, it is enhanced with respect to the dye film alone.

The effect of the photonic band structure on the microcavity optical spectra (both transmittance and fluorescence) is further confirmed through angle-resolved measurements.^46^Figure 5c,d shows the two sets of data as contour plots. On the *y*-axis, the collection angle for photoluminescence (Figure 5c) and transmittance (Figure 5d) against the wavelength (*x*-axis) is shown. Transmittance and photoluminescence values for each angle–wavelength pair are shown as color tones, with stronger emission and transmittance represented in lighter yellowish tones. In Figure 5d, the photon bandgap (the dark-blue, low-transmittance region) and the cavity mode (highlighted by the dotted line) shift toward shorter wavelengths, increasing the angle of incidence. This blueshift is the standard behavior for photonic structures of this kind, such as DBRs as well as MCs, as reported in many references.^30,47^ The trend of the cavity mode, highlighted by the dashed black line, is closely mirrored by the change in the MC’s photoluminescence upon angle increase. Indeed, the peak in photoluminescence (bright yellow band in Figure 5c, highlighted by the black dashed line) is subjected to the same blueshift.^47^ This behavior further confirms that the MC is redistributing the emission according to the spectral position of its cavity modes, enhancing the signal in their correspondence and suppressing it in correspondence to the photon bandgap. Additionally, around 35°, when the longer wavelength cavity mode has shifted enough to match the dye photoluminescence spectrum, the emission is accordingly increased in its correspondence. Overall, these measurements indicate that the structure is performing as expected from a typical polymer MC.

We performed radiative rate measurements on the microcavity, as reported in the Supporting Information S8. There, we observed a change in the radiative rate due to the varied dielectric environment with respect to air. In general, all of the results obtained regarding microcavity fabrication—apart from fine effects on the radiative rate—testify that the dip-coating microcavity growth process allows one to match the precision required to obtain an optical-quality microcavity.

## Conclusions

This work unambiguously demonstrates the successful conversion of a commercial FDM 3D printing system into a dip-coating system able to fabricate photonic structures such as DBRs and planar microcavity. Two polymers with low and high refractive indexes, namely, AQ and PVK, were used as building materials for DBRs. The dip-coating is determined by a delicate balance of factors, including the viscosity of the solution, withdrawal speed, drying conditions, and substrate properties. As a preliminary study, the withdrawal speed and drying conditions were used to optimize the fabrication process of one-dimensional photonic crystals. The work carried out has confirmed that the withdrawal speed influences the periodicity of the structure (i.e., the thickness of the layers). The drying conditions, such as the uniform heating of the substrate, have an influence on the homogeneity of the thickness of the layers forming the crystal and therefore on the homogeneity of the optical response across the whole surface. Reproducibility of the working conditions was demonstrated through repeated fabrications. To push the limits of the system, a delicate photonic structure (the planar microcavity) was fabricated, partly with recycled materials. Its optical characterization showed the characteristic features well reported in the literature of this kind of microcavity, as well as their effect on the photoluminescence of the embedded dye. The similarity of the microcavity’s photonic structure with that of spin-coated samples is further confirmed by angle-resolved transmission and photoluminescence measurements, which show the typical shift of the photon bandgap and the microcavity mode to shorter wavelengths as the collection angle is increased. We believe the results obtained with this experimental method shed light on the intricate details of the dip-coating process of all-polymer photonic structures and will serve as a basis for further research and refinement in this field.

## Supporting Information Available

Thermal video (MP4)
Picture of the experimental setup (S1); pictures of the spoon (S2); reflectance spectra and digital images of three DBRs fabricated in three copies each with the converted 3D-printed (S3a–c); AFM measurements of a dip- and spin-coated sample (S4); IR spectra of the drop-cast cavity film and the reference polystyrene (S5); DBR constituting the planar microcavity (S6); normalized reflectance spectrum of the microcavity (S7); and results of the lifetime measurements (S8) (PDF)

## References

[ref1] Scriven L. E. Physics and Applications of DIP Coating and Spin Coating. MRS Online Proc. Libr. 1988, 121 (1), 717–729. 10.1557/PROC-121-717.

[ref2] Grosso D. How to Exploit the Full Potential of the Dip-Coating Process to Better Control Film Formation. J. Mater. Chem. A 2011, 21 (43), 17033–17038. 10.1039/c1jm12837j.

[ref3] Ossila. Dip Coating Guide. 2023. www.ossila.com/en-eu/pages/dip-coating (accessed September 2023).

[ref4] Brinker C. J. Dip Coating. In Chemical Solution Deposition of Functional Oxide Thin Films; Schneller T.; Waser R.; Kosec M.; Payne D., Eds.; Springer Vienna, 2013; pp 233–261.

[ref5] Yimsiri P.; Mackley M. R. Spin and Dip Coating of Light-Emitting Polymer Solutions: Matching Experiment with Modelling. Chem. Eng. Sci. 2006, 61 (11), 3496–3505. 10.1016/j.ces.2005.12.018.

[ref6] Goyal M.; Singh K.; Bhatnagar N. Conductive Polymers: A Multipurpose Material For Protecting Coating. Prog. Org. Coat. 2024, 187, 108083 10.1016/j.porgcoat.2023.108083.

[ref7] Xu Z.; Lu J.; Zhang G.; Liu R.; Zhang W.; Meng Q. Compact Sioc Ceramic Composite Nanofiltration Membranes by Slow Dip Coating for Water Purification. Ceram. Int. 2024, 50 (13, Part A), 23115–23123. 10.1016/j.ceramint.2024.04.034.

[ref8] Jebali S.; Vayer M.; Belal K.; Mahut F.; Sinturel C. Dip-Coating Deposition of Nanocomposite Thin Films Based on Water-Soluble Polymer And Silica Nanoparticles. Colloids Surf., A 2024, 680, 132688 10.1016/j.colsurfa.2023.132688.

[ref9] Aksakal B.; Hanyaloglu C. Bioceramic Dip-Coating on Ti–6Al–4V and 316L SS Implant Materials. J. Mater. Sci.: Mater. Med. 2008, 19 (5), 2097–2104. 10.1007/s10856-007-3304-2. 17968501

[ref10] Jafri N. N. M.; Jaafar J. 5 - Dip Coating Technique. In Advanced Ceramics For Photocatalytic Membranes; Othman M. H. D.; Rahman M. A.; Matsuura T.; Adam M. R.; Mohd Makhtar S. N. N., Eds.; Elsevier, 2024; pp 101–127.

[ref11] Tahir Z.; Rashid M. U.; Kim S.; Park Y. C.; Tran H. N.; Cho S.; Kim Y. S. Highly Reflective Distributed Bragg Reflectors for Planar microcavities: From Modelling to Experimentation. Trans. Electr. Electron. Mater. 2024, 25 (1), 32–39. 10.1007/s42341-023-00483-3.

[ref12] Ge A.; Sun L.; Xie M.; Cui H.; Zhou D.; Ma L.; Zhang X.; Huan Y.; Tian H.; Jing W.; et al. High Quality Near-Infrared Single-Mode Lasing from γ-InSe Using a Transferrable Planar Microcavity. Adv. Opt. Mater. 2024, 12 (17), 2303286 10.1002/adom.202303286.

[ref13] Zhu H.; He Z.; Wang J.; Zhang W.; Pei C.; Ma R.; Zhang J.; Wei J.; Liu W. Microcavity Complex Lasers: from Order to Disorder. Ann. Phys. 2024, 536 (9), 2400112 10.1002/andp.202400112.

[ref14] Ishii T.; Pérez-Sánchez J. B.; Yuen-Zhou J.; Adachi C.; Hatakeyama T.; Kéna-Cohen S. Modified Prompt and Delayed Kinetics in a Strongly Coupled Organic Microcavity Containing a Multiresonance TADF Emitter. ACS Photonics 2024, 11 (10), 3998–4007. 10.1021/acsphotonics.4c00488.

[ref15] Lova P.; Manfredi G.; Comoretto D. Advances in Functional Solution Processed Planar 1D Photonic Crystals. Adv. Opt. Mater. 2018, 6 (24), 1800730 10.1002/adom.201800730.

[ref16] Palo E.; Papachatzakis M. A.; Abdelmagid A.; Qureshi H.; Kumar M.; Salomäki M.; Daskalakis K. S. Developing Solution-Processed Distributed Bragg Reflectors for Microcavity Polariton Applications. J. Phys. Chem. C 2023, 127 (29), 14255–14262. 10.1021/acs.jpcc.3c01457. PMC10388359 37529668

[ref17] Barton I.; Matejec V.; Mrazek J.; Predoana L.; Zaharescu M. Properties of Silica and Silica-Titania Layers Fabricated from Silica Sols Containing Fumed Silica. Opt Mater. 2018, 77, 187–197. 10.1016/j.optmat.2018.01.037.

[ref18] Yuehui W.; Xing Y. High-Reflection Optical Thin Films Based on SiO_2_/TiO_2_ Nanoparticles Multilayers by Dip Coating. Micro Nano Lett. 2018, 13 (9), 1349–1351. 10.1049/mnl.2018.0045.

[ref19] Bachevillier S.; Yuan H. K.; Strang A.; Levitsky A.; Frey G. L.; Hafner A.; Bradley D. D. C.; Stavrinou P. N.; Stingelin N. Fully Solution-Processed Photonic Structures from Inorganic/Organic Molecular Hybrid Materials and Commodity Polymers. Adv. Funct. Mater. 2019, 29 (21), 1808152 10.1002/adfm.201808152.

[ref20] Joannopoulos J. D.; Johnson S. G.; Winn J. N.; Meade R. D. Photonic Crystals: Molding the Flow of Light; Princeton University Press, 1995.

[ref21] Skorobogatiy M.; Yang J. Fundamentals of Photonic Crystal Guiding; Cambridge University Press, 2009.

[ref22] Yablonovitch E. Inhibited Spontaneous Emission in Solid-State Physics and Electronics. Phys. Rev. Lett. 1987, 58 (20), 2059–2062. 10.1103/PhysRevLett.58.2059. 10034639

[ref23] John S. Strong Localization of Photons in Certain Disordered Dielectric Superlattices. Phys. Rev. Lett. 1987, 58 (23), 2486–2489. 10.1103/PhysRevLett.58.2486. 10034761

[ref24] Yang Y.; Yu L.; Jiang X.; Li Y.; He X.; Chen L.; Zhang Y. Recent Advances in Photonic Crystal-Based Chemical Sensors. Chem. Commun. 2024, 60 (69), 9177–9193. 10.1039/D4CC01503G. 39099372

[ref25] Shi T.; Kou D.; Gao L.; Xue Y.; Zhang S.; Ma W. One-Dimensional Responsive Photonic Crystals Assembled by Polymer Nanogels and TiO_2_ Nanoparticles for Rapid Detection of Glucose. ACS Appl. Nano Mater. 2024, 7 (3), 3116–3128. 10.1021/acsanm.3c05440.

[ref26] Sampath D.; Narasimhan V. One-Dimensional Defect Layer Photonic Crystal Sensor for Purity Assessment of Organic Solvents. ACS Omega 2024, 9 (8), 9625–9632. 10.1021/acsomega.3c09589. 38434907 PMC10905966

[ref27] Zheng W.; Zhang N.; Murtaza G.; Meng Z.; Wu L.; Qiu L. Naked-Eye Visual Thermometer Based on Glycerol—Nonclose-Packed Photonic Crystals for Real-Time Temperature Sensing and Monitoring. ACS Appl. Mater. Interfaces 2024, 16 (10), 13041–13051. 10.1021/acsami.3c17566. 38417142

[ref28] Lanfranchi A.; Megahd H.; Lova P.; Comoretto D. Engineering All-Polymer Planar Photonic Crystals as Aegises Against Sunlight Overheating. Chem. Eng. Sci. 2024, 283, 119377 10.1016/j.ces.2023.119377.

[ref29] Escher A.; Megahd H.; Tavella C.; Comoretto D.; Lova P. Colorimetric Polymer Sensors for Smart Packaging. Macromol. Chem. Phys. 2023, 224 (14), 2300022 10.1002/macp.202300022.

[ref30] Megahd H.; Lova P.; Sardar S.; D’Andrea C.; Lanfranchi A.; Koszarna B.; Patrini M.; Gryko D. T.; Comoretto D. All-Polymer microcavities for the Fluorescence Radiative Rate Modification of a Diketopyrrolopyrrole Derivative. ACS Omega 2022, 7 (18), 15499–15506. 10.1021/acsomega.2c00167. 35571840 PMC9096937

[ref31] Vahala K. J. Optical microcavities. Nature 2003, 424 (6950), 839–846. 10.1038/nature01939. 12917698

[ref32] Noda S.; Fujita M.; Asano T. Spontaneous-Emission Control by Photonic Crystals And Nanocavities. Nat. Photonics 2007, 1 (8), 449–458. 10.1038/nphoton.2007.141.

[ref33] Yang L.; Ma X.; Long T.; Huang H.; Ren J.; Gu C.; An C.; Liao B.; Fu H.; Liao Q. Dual-Wavelength Exciton-Polariton Condensation via Relaxation of Multiple Vibrational Quanta in Organic microcavities. ACS Photonics 2024, 11 (11), 4700–4706. 10.1021/acsphotonics.4c01195.

[ref34] Ossila. Spin Coating Guide. 2023. www.ossila.com/pages/spin-coating (accessed September 2023).

[ref35] Ossila. Dip Coater. www.ossila.com/products/dip-coater?variant=12376293376117 (accessed September 2023).

[ref36] Holmarc. Multiple Dip Coater. www.holmarc.com/multiple_dip_coater.php (accessed March 2024).

[ref37] Rauh F.; Bienek O.; Sharp I. D.; Stutzmann M. Conversion of a 3D printer for versatile automation of dip coating processes. Rev. Sci. Instrum. 2023, 94 (8), 083901 10.1063/5.0128116. 37534978

[ref38] 3DPrinterAcademy. What is G-code? www.3dprinteracademy.com/blogs/basics/what-is-g-code (accessed September 2023).

[ref39] Girelli A.; Leno M.; Parisi F. Trattato di Chimica Industriale e Applicata; Zanichelli, 1969.

[ref40] Gordon P. F.; Gregory P. Organic Chemistry in Colour; Springer Science & Business Media, 2012.

[ref41] Ooyama Y.; Yagi S. Progress in the Science of Functional Dyes; Springer Nature, 2021.

[ref42] Landau L.; Levich B. Dragging of a Liquid by a Moving Plate. In Dynamics of Curved Fronts; Academic Press, 1988; pp 42–54.

[ref43] Unger K.; Resel R.; Czibula C.; Ganser C.; Teichert C.; Jakopic G.; Canazza G.; Gazzo S.; Comoretto D. Distributed Bragg Reflectors: Morphology of Cellulose Acetate and Polystyrene Multilayers. In 2014 16th International Conference on Transparent Optical Networks (ICTON), 6–10 July 2014; IEEE, 2014; pp 1–4.

[ref44] Toluene. https://webbook.nist.gov/cgi/cbook.cgi?ID=C108883&Mask=4 (accessed February 2025).

[ref45] Ethanol. https://webbook.nist.gov/cgi/cbook.cgi?ID=C64175&Units=SI&Mask=4#Thermo-Phase (accessed February 2025).

[ref46] Barth M.; Gruber A.; Cichos F. Spectral and Angular Redistribution of Photoluminescence Near a Photonic Stop Band. Phys. Rev. B 2005, 72 (8), 085129 10.1103/PhysRevB.72.085129.

[ref47] Megahd H.; Villarreal Brito M.; Lanfranchi A.; Stagnaro P.; Lova P.; Comoretto D. Control of Near-Infrared Dye Fluorescence Lifetime in All-Polymer microcavities. Mater. Chem. Front. 2022, 6 (17), 2413–2421. 10.1039/D2QM00313A.

