# Supplementary material for: Dip-Coating Fabrication of All-Polymer Multilayer Photonic Crystals through 3D Printer Conversion
Source: ACS Appl Polym Mater. 2025 Apr 9;7(8):4779–86. doi: 10.1021/acsapm.4c04077 (PMC12039967; doi:10.1021/acsapm.4c04077)
Supplement: Supplementary file 2 — ap4c04077_si_002.pdf [file ap4c04077_si_002.pdf]

Supporting Information for the Article:

Dip Coating Fabrication Of All-Polymer Multilayer Photonic Crystals Through 3D Printer Conversion

Martina Martusciello[1], Coralie Hervieu[1,2], Daniela Di Fonzo[1], Andrea Lanfranchi[1], Paola Lova[1], Davide Comoretto[1].*

[1] Dipartimento di Chimica e Chimica Industriale, Università di Genova, via Dodecaneso 31, 16146 Genova (Italy)

[2] École d'Ingénieurs Publique du MESRI, Sigma Clermont, 20 Avenue Blaise Pascal, TSA 62006, 63178 Aubiere Cedex

davide.comoretto@unige.it

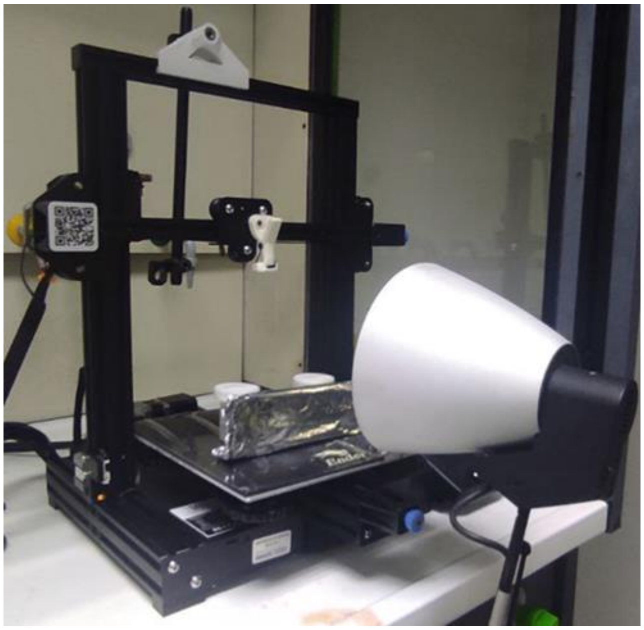

Figure S1. Picture of the experimental setup.

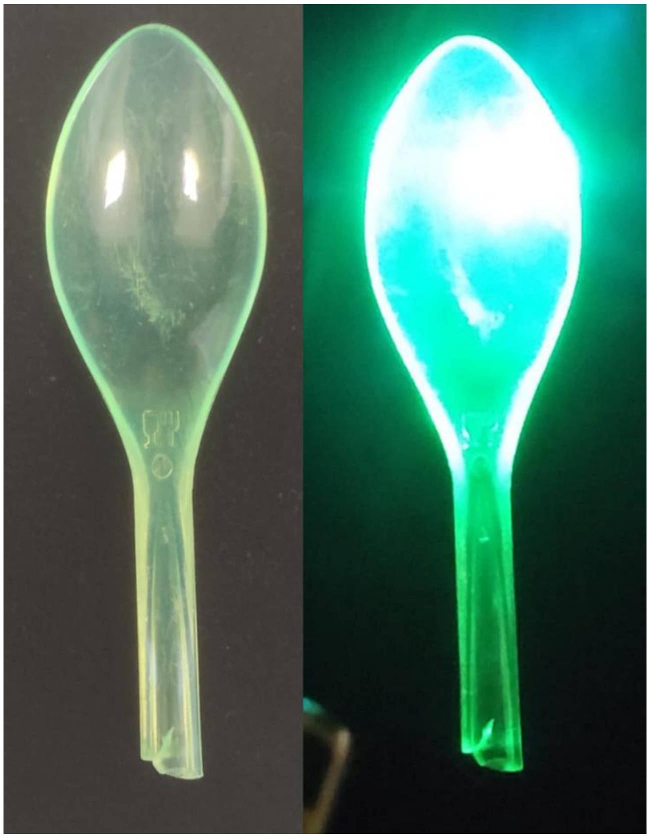

Figure S2. Picture of the spoon under white light and 405 nm laser irradiation.

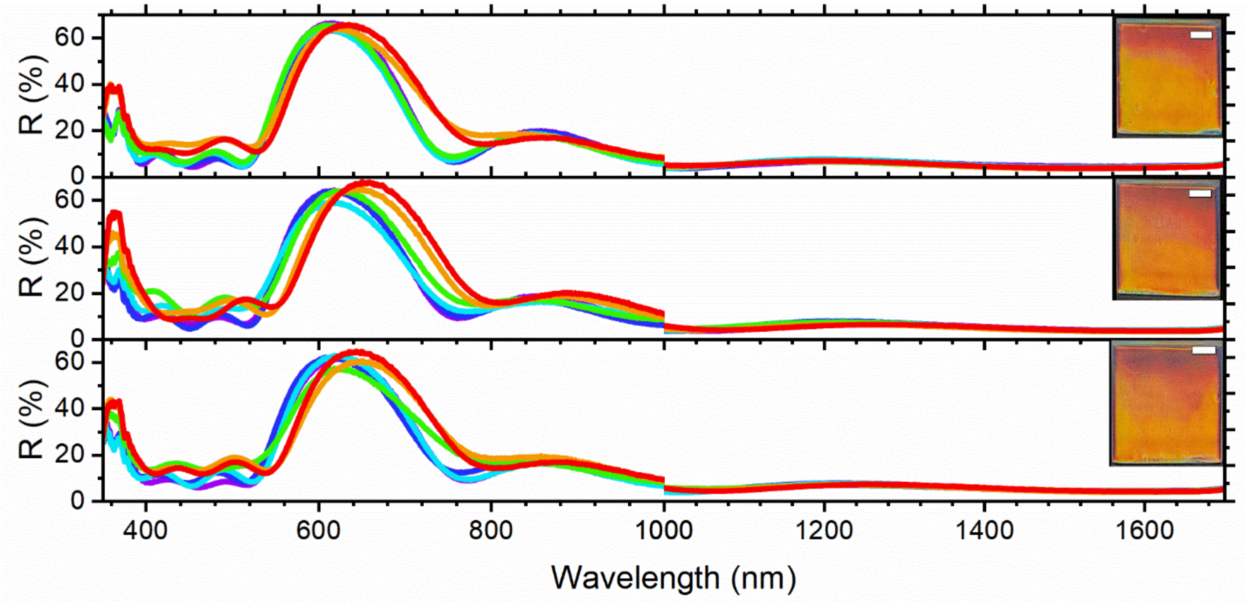

Figure S3a. Reflectance spectra and digital image (inset) of three DBRs produced in the same conditions (PVK 10 mg ml^{-1}, AQ 1:20, 3 mm s^{-1}, 250 W, 4.5 bilayers). The white dash is a 5 mm scale.

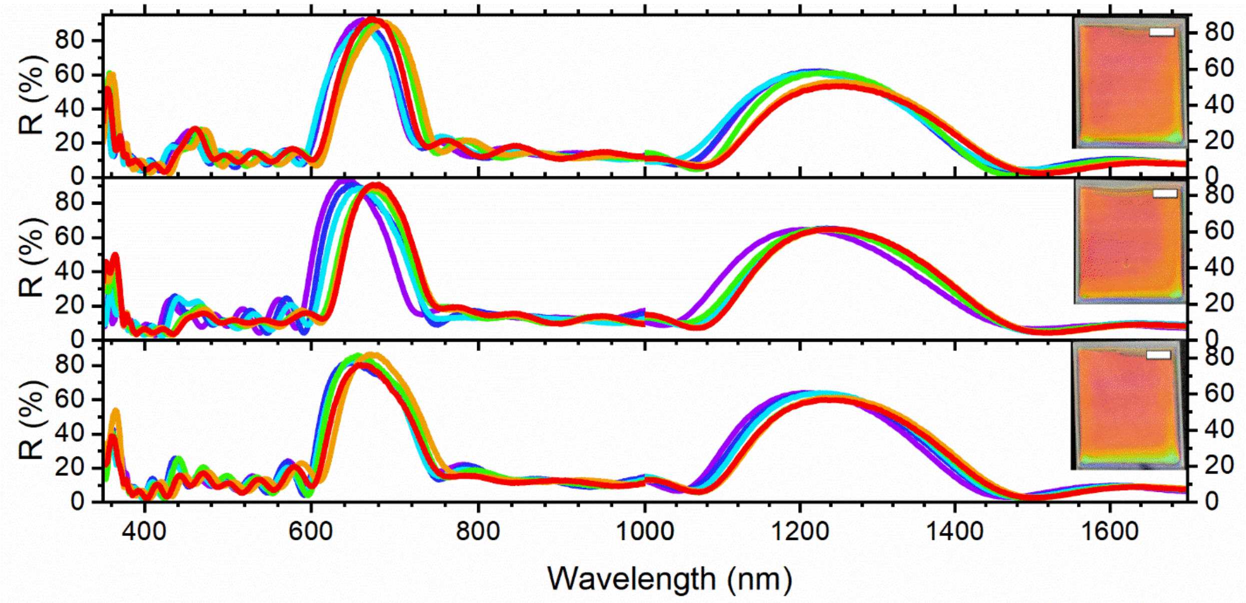

Figure S3b. Reflectance spectra and digital image (inset) of three DBRs produced at the same conditions (PVK 10 mg ml^{-1}, AQ 1:10, 4 mm s^{-1}, 250 W, 4.5 bilayers). The white dash is a 5 mm scale.

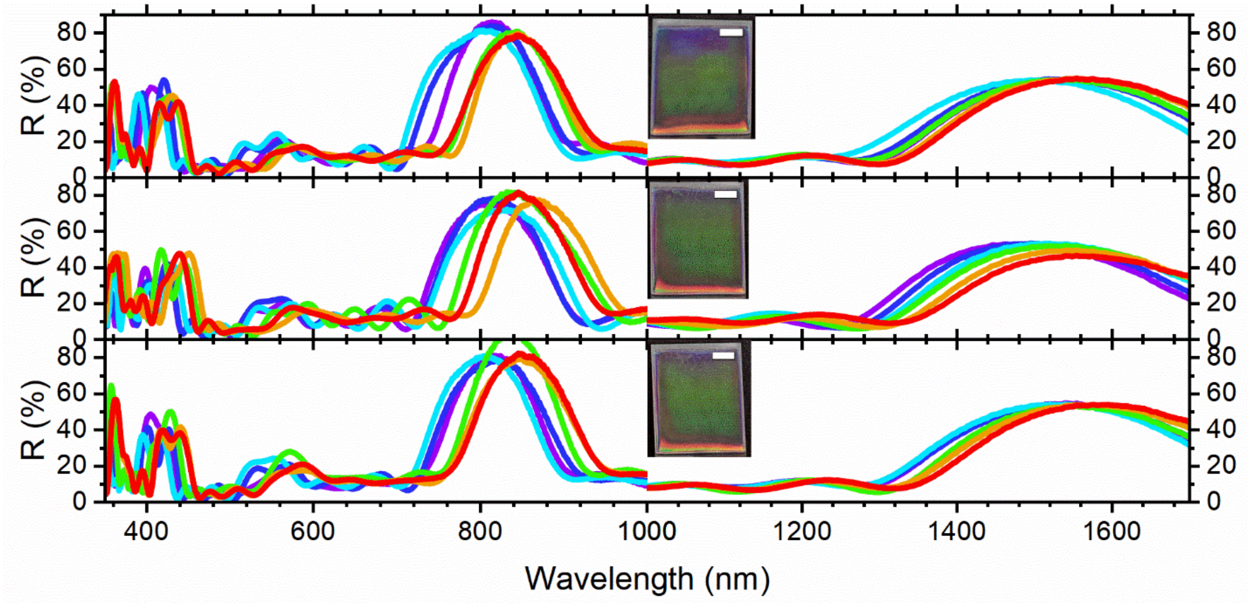

Figure S3c. Reflectance spectra and digital image (inset) of three DBRs produced at the same conditions (PVK 11 mg ml^{-1}, AQ 1:10, 6 mm s^{-1} 250 W, 4.5 bilayers). The white dash is a 5 mm scale.

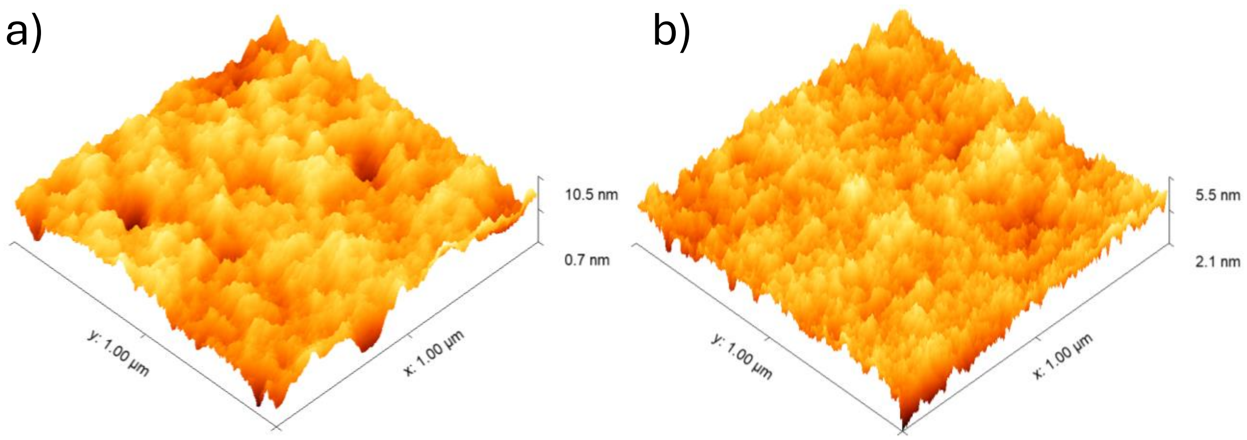

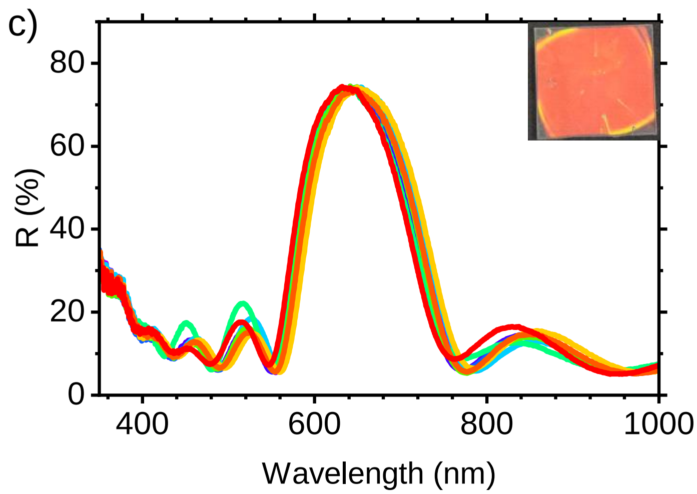

Figure S4. Atomic Force Microscope micrographs of a 1x1 μm^2 measurements of **a)** the dip-coated DBR S3a, second from top, and **b)** a spin-coated DBR with the same number of layers and similar structure. **c)** Reflectance spectra over 9 points (from the top left) of the spin-coated DBR and its picture (in the inset).

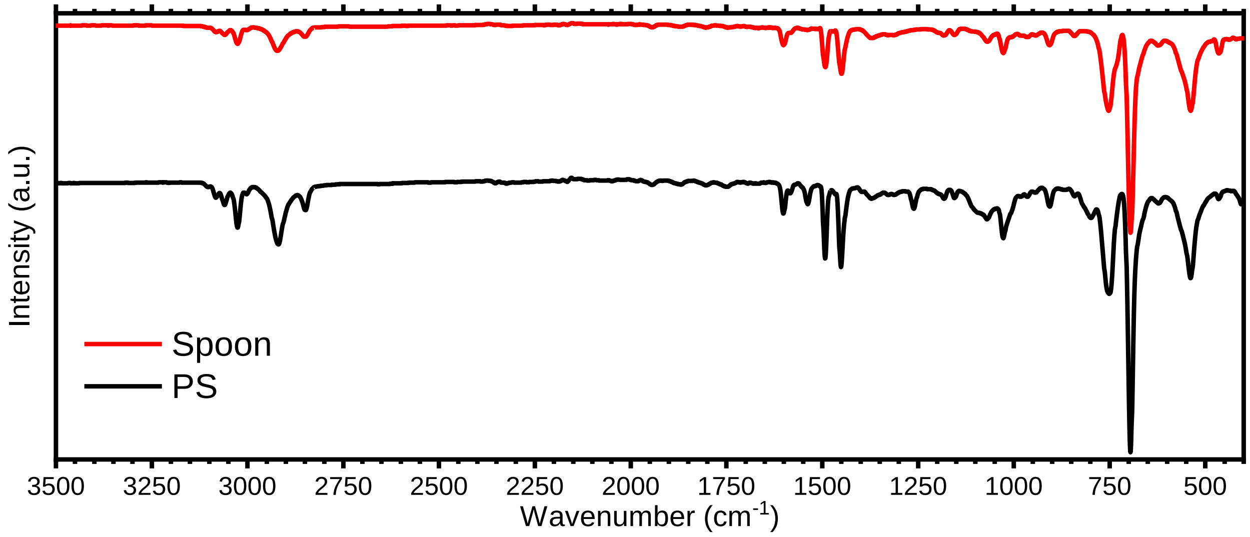

Figure S5. IR spectra of the drop cast cavity film (red) and reference polystyrene (black).

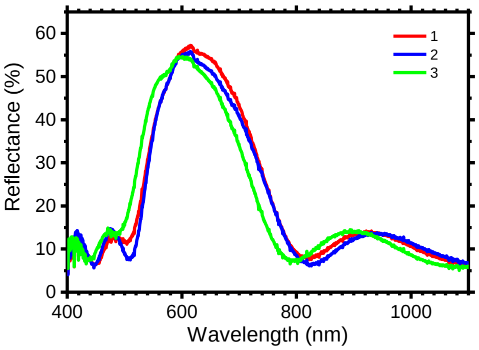

Figure S6. Reflectance spectra collected in three locations of the DBR before depositing the defective layer.

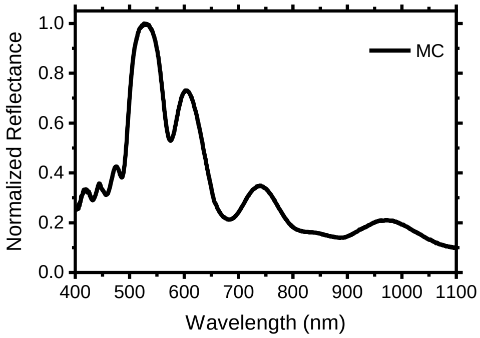

Figure S7. Normalized reflectance spectra of the MC.

Section S8. A final additional test on the cavity quality concerns the emission lifetime of the dye. Indeed, the radiative rate of an emitter is not only dictated by its intrinsic properties, but also by the dielectric environment. To verify this effect, Figure S8 reports the fluorescence decay upon excitation with a pulsed laser (λ = 405 nm) for the standalone defect film (blue), the microcavity (black) and compares them with the Instrument Response Function (IRF, light gray). A remarkable difference between the decays of MC with respect to the dye itself can clearly be observed. Focusing especially on the timeframe after 4 ns, this indicates the dielectric environment and the confinement provided by the high-dielectric contrast DBR does affect the fluorescence decay

processes, as reported previously in these systems.[1] At this point, a more in-depth study would be needed to provide more quantitative considerations; however, this would be beyond the scope of the paper. On a qualitative level, indeed, the data are in sufficient agreement with what previously observed in similar systems[1] to suggest the dip coating system here presented allows to fabricate functional all-polymer optical MCs.

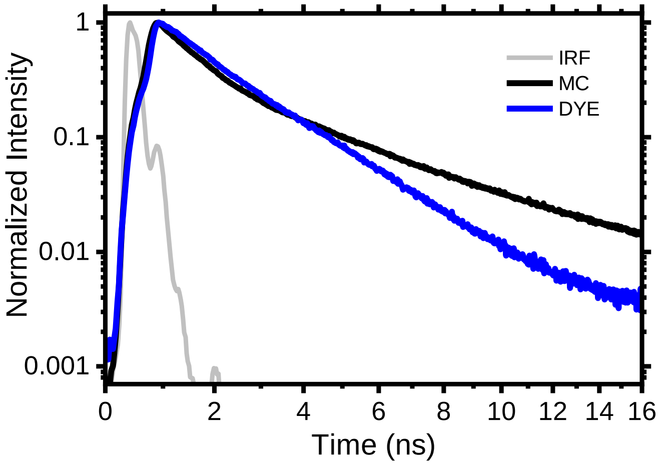

Figure S8. Comparison of the fluorescence decay upon excitation with a pulsed laser (λ=405nm) for the standalone defect film (blue), MC (black), and the IRF (gray) in a log-log plot.

References

(1) Megahd, H.; Lova, P.; Sardar, S.; D'Andrea, C.; Lanfranchi, A.; Koszarna, B.; Patrini, M.; Gryko, D. T.; Comoretto, D. All-Polymer Microcavities for the Fluorescence Radiative Rate Modification of a Diketopyrrolopyrrole Derivative. *ACS Omega* **2022**, *7* (18), 15499-15506. DOI: 10.1021/acsomega.2c00167.
